# Zno Micro/Nanostructures Grown on Sapphire Substrates Using Low-Temperature Vapor-Trapped Thermal Chemical Vapor Deposition: Structural and Optical Properties

**DOI:** 10.3390/ma11010003

**Published:** 2017-12-21

**Authors:** Po-Sheng Hu, Cheng-En Wu, Guan-Lin Chen

**Affiliations:** 1 Institute of Photonic System, National Chiao Tung University, Tainan City 71150, Taiwan; domo4413@gmail.com; 2 Institute of Imaging and Biomedical Photonics, National Chiao Tung University, Tainan City 71150, Taiwan; wu71789012@yahoo.com.tw

**Keywords:** low temperature process, vapor-trapped CVD, structural and optical characterization

## Abstract

In this research, the Zn(C_5_H_7_O_2_)_2_·xH_2_O-based growth of ZnO micro/nanostructures in a low temperature, vapor-trapped chemical vapor deposition system was attempted to optimize structural and optical properties for potential biomedical applications. By trapping in-flow gas molecules and Zinc vapor inside a chamber tube by partially obstructing a chamber outlet, a high pressure condition can be achieved, and this experimental setup has the advantages of ease of synthesis, being a low temperature process, and cost effectiveness. Empirically, the growth process proceeded under a chamber condition of an atmospheric pressure of 730 torr, a controlled volume flow rate of input gas, N_2_/O_2_, of 500/500 Standard Cubic Centimeters per Minute (SCCM), and a designated oven temperature of 500 °C. Specifically, the dependence of structural and optical properties of the structures on growth duration and spatially dependent temperature were investigated utilizing scanning electron microscopy, X-ray diffraction (XRD), photoluminescence (PL), and ultraviolet-visible transmission spectroscopy. The experimental results indicate that the grown thin film observed with hexagonal structures and higher structural uniformity enables more prominent structural and optical signatures. XRD spectra present the dominant peaks along crystal planes of (002) and (101) as the main direction of crystallization. In addition, while the structures excited with laser wavelength of 325 nm emit a signature radiation around 380 nm, an ultraviolet lamp with a wavelength of 254 nm revealed distinctive photoluminescence peaks at 363.96 nm and 403.52 nm, elucidating different degrees of structural correlation as functions of growth duration and the spatial gradient of temperature. Transmittance spectra of the structures illustrate typical variation in the wavelength range of 200 nm to 400 nm, and its structural correlation is less significant when compared with PL.

## 1. Introduction 

ZnO is a popular II-VI N-type semiconductor material composed of hexagonal wurtzite structure and has a melting temperature of 1975 °C, enabling good thermal stability [1]. Its direct energy gap and exciton binding energy at room temperature are 3.37 eV and 60 meV, respectively, making it highly suitable for optoelectronic applications [2,3,4,5], and more recently, for the detection of gas and biological molecules, attributing to their unique electrochemical properties [6,7,8].

Many current chemical and physical methods have been applied for the synthesis of ZnO structures [9,10]. On the side of chemical vapor deposition (CVD), many variants of it have criteria including special molecules, plasma or high temperature [11,12]. Among thermal CVD methods, most of the grown ZnO nanostructures are based on ZnO powder or its mixture with graphite to reduce the melting point [5]. Also, Liu et al. suggested vaporization of ZnO powder through an indirect rise of temperature by containing it in a capped crucible [13]. However, the lowest required temperature of such a process is still around 900 °C, rendering small amounts of available Zinc vapor, which makes it difficult for material to deposit on the substrate, and requires a thin layer of metallic coating to facilitate the growth of the nanostructure [6]. 

Alternatively, previous research explored a precursor, Zinc acetylacetonate hydrate, Zn(C_5_H_7_O_2_)_2_·xH_2_O, that requires a very low melting temperature of 130 °C to 140 °C, allows more zinc vapor for direct deposition on bare substrates, and has been used to synthesize the nanostructures of whisker, film and pillars [7,8,14]. The applied chamber pressure for growing ZnO structures is induced by a high vacuum condition alongside a constant in-flow of selected gas, rendering the resultant pressure close to atmospheric pressure, 200 torr. Contrary to the synthetic process under constant gas flow, results reported by Chang et al. exploited an air-trapping technique that captures the reaction gas and ZnO vapor inside a glass bottle where a few substrates were placed at different locations for material deposition [15].

In the research presented herein, the growth of ZnO micro/nanostructures using Zinc acetylacetonate hydrate in a two-heating zone, air-trapped thermal CVD with designated oven temperature of 500 °C and chamber pressure of 760 torr, which was produced by a combination of in-flow of O_2_ and N_2_ with a partial obstruction of the chamber’s output valve, was attempted. Additionally, the structural and optical properties of the structures as functions of growth duration, 1 h, 3 h and 6 h, and spatially dependent temperature were characterized and analyzed in search of a suitable growth condition that enables material characteristics for potential biomedical applications, such as to simultaneously identify and isolate cancer cells on a substrate.

From this work, several conclusions were drawn. As the growth duration increases, the feature sizes of the grown structures increase, and the result of crystallization tends to produce a film-like structure. Also, as the substrate position gets closer to the center of the high temperature oven, both density and uniformity of the shapes of the structures are lowered, and the organization of the overall structures is randomized. Additionally, the results of XRD and 325 nm wavelength-induced photoluminescence (PL) spectra present stronger signals of stereoscopic over planar thin film-like structures, most of which were grown with the duration of 3 h and 6 h, respectively. In contrast, PL intensities induced by the wavelength of 254 nm and transmittance have a poor structural correlation.

## 2. Methods and Materials

### 2.1. Vapor-Trapped Thermal CVD

**Figure 1:**
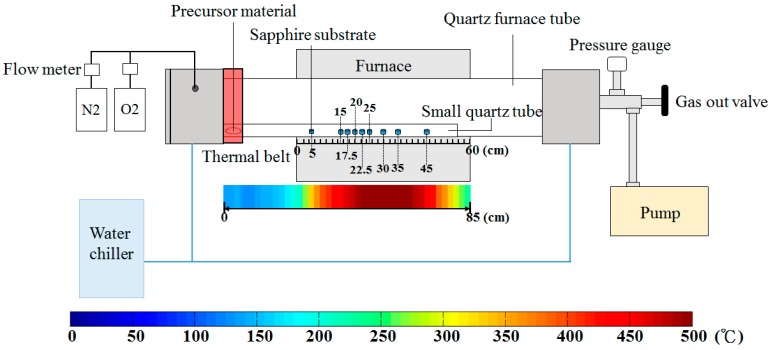
Schematic diagram of custom-built vapor-trapped CVD and a color bar for presentation of temperature distribution. The overall furnace zone has a length of 60 cm, and the substrates were aligned at 5 cm, 15 cm, 17.5 cm, 20 cm, 22.5 cm, 25 cm, 30 cm, 35 cm and 45 cm indicated by tic marks on a ruler. The color bar presents the spatial distribution of temperature along the distance from the thermal belt to the end of furnace zone, which has a total length of 85 cm.

Figure 1 illustrates a schematic diagram of a two-heating zone, vapor-trapped thermal CVD, and a color bar that indicates the spatial distribution of temperature along the length from a thermal belt to furnace zone. Both ends of the quartz furnace tube are sealed-well by stainless steel envelopes. To proceed with the experiment, the quartz furnace tube chamber is pumped by a mechanical vacuum pump until the pressure inside the chamber reaches the zone of the vacuum condition with pressure set to about 7 × 10^−3^ torr. A gas inlet near the left stainless envelope allows the respective carrier and reaction gas, N_2_ and O_2_, with flow rates of 500 SCCM to be introduced into the quartz furnace tube. Alongside the gas flow, by adjusting a manual outlet valve on the right hand side of the quartz tube, the chamber pressure can be set to 1 atmospheric pressure. Subsequently, once the designated oven temperature of 500 °C was reached, the thermal belt was set immediately to 140 °C for vaporizing the ZnO precursor, Zn(C_5_H_7_O_2_)_2_·xH_2_O, which is the commencement time-point of the duration of structural growth on the substrates. After the material deposition is finished, the oven, thermal belt and gas flow are switched off for cooling. 

### 2.2. Sample Preparation 

To avoid potential contamination of unrelated materials, the growth of the ZnO structures is carried out by loading a small quartz tube with a diameter of 1.5 cm and a length of 80 cm containing a ZnO precursor material, Zinc acetylacetonate hydrate (Zn(C_5_H_7_O_2_)_2_·xH_2_O, Sigma Aldrich, St. Louis, MO, USA, 98%) and (0001) sapphire substrates with dimension of 1.5 × 1.5 cm^2^ into the furnace quartz tube. The precursor (250 mg) is lined up along the extent of the thermal belt, and the substrates were aligned with tic marks of 5 cm, 15 cm, 17.5 cm, 20 cm, 22.5 cm, 25 cm, 30 cm, 35 cm and 45 cm as shown in the ruler of Figure 1.

### 2.3. X-ray Diffraction

XRD (D2 Phaser, Bruker AXS Gmbh, Karlsruhe, Germany) was used to identify the lattice structure of ZnO micro/nanostructures and to examine the dependence of resultant crystallization on the growth duration and position-dependent temperature, In the preparation of the XRD experiment, The ZnO structure grown on a substrate was placed on the quartz boat inside the XRD chamber for examination with the scanning angle ranging from 30° to 80°, within which the pronounced diffraction peaks occur, according to the Joint Committee on Powder Diffraction Standards (JCPDS) card No. 36-1451, and the scanning rate is 4° per min. 

### 2.4. Scanning Electron Microscopy

A thermal field emission scanning electron microscope (JSM-7000F, FE-SEM, JEOL Tokyo, Japan) was used to observe the surface structure of the grown structures. The sample was fixed on the stage inside the SEM chamber, and with the vacuum turned on for some time, an electron beam is introduced and focused onto the sample. Images with magnification of 10,000× and 50,000× were acquired.

### 2.5. Photoluminescence

In this research, an He-Cd laser operating at a wavelength of 325 nm, and ultraviolet (UV) lamp emitting photon (UVGL-25, UVP, Upland, CA, USA) with wavelength of 254 nm were used as the light sources for photoexcitation to acquire PL spectra and address how changes in ZnO structures play a role in manipulating the anticipated PL peaks. To prepare for the PL experiment, the light sources, including laser beam or lamp light, are directed onto the surfaces of sample substrates for data acquisition. The analysis of PL spectra allows assessment of the changes of material structure, process of crystallization, and defects.

### 2.6. Transmittance Spectroscopy

An ultraviolet-visible (UV-VIS) spectrophotometer (UV-1800 SHIMADZU, Kyoto, Japan) was used to measure the transmittance spectra, ranging from 190 nm to 1100 nm. A light source directly illuminates the surface of the sample substrates placed on a stage with a hole in the middle where the transmitted light can be detected by a spectrometer located beneath the stage. The resultant spectral signal can then be detected by the spectrometer for data analysis.

## 3. Results 

**Figure 2:**
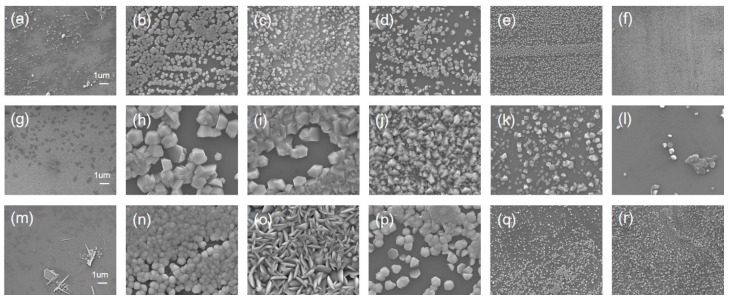
SEM images of the ZnO micro/nanostructures grown on (0001) sapphire substrates. For the growth duration of (**a**–**f**) 1 h, (**g**–**l**) 3 h, (**m**–**r**) 6 h, the structures grown at (**a**,**g**,**m**) 5 cm, (**b**,**h**,**n**) 15 cm, (**c**,**i**,**o**) 17.5 cm, (**d**,**j**,**p**) 20 cm, (**e**,**k**,**q**) 22.5 cm, (**f**,**l**,**r**) 25 cm are illustrated. Magnification is 10,000×.

**Figure 3:**
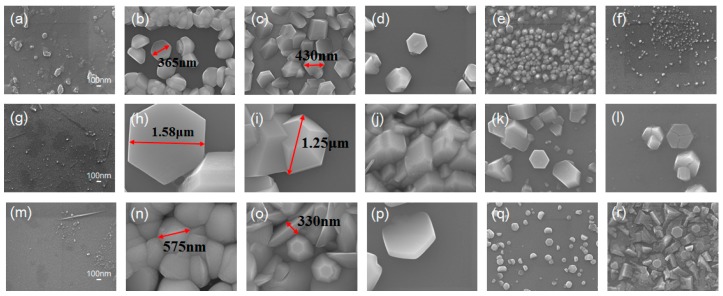
SEM images of the ZnO micro/nanostructures grown on (0001) sapphire substrates. For the growth duration of (**a**–**f**) 1 h, (**g**–**l**) 3 h, (**m**–**r**) 6 h, the structures grown at (**a**,**g**,**m**) 5 cm, (**b**,**h**,**n**) 15 cm, (**c**,**i**,**o**) 17.5 cm, (**d**,**j**,**p**) 20 cm, (**e**,**k**,**q**) 22.5 cm, (**f**,**l**,**r**) 25 cm are illustrated. Magnification is 50,000×.

Figure 2 illustrates 10,000× SEM images of ZnO structures grown with corresponding duration of 1 h, 3 h and 6 h on top, middle and bottom rows. As can be seen from the figure, the vapor-trapped condition in conjunction with the temperature gradient along the length of furnace tube promotes growth of the structures with high uniformity and density at the position of 15 cm, 17.5 cm, 20 cm. Close-up SEM images with magnification of 50,000× are presented in Figure 3. Formation of substantial structures occurred beyond the position of 15 cm; Figure 3b,h associated with duration of 1 h and 3 h can be observed with complete ZnO hexagonal structures with feature size ranging from 300 nm to 400 nm and 1 μm to 2 μm as indicated by arrow lines in the figures. Figure 3n was observed with a collection of jammed granular pieces with a range of feature size from 400 nm to 600 nm.

**Figure 4:**
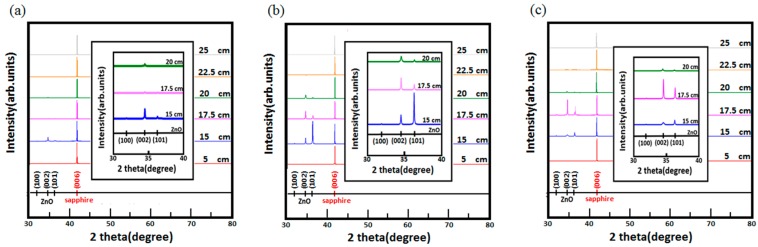
XRD spectra of the ZnO micro/nanostructures grown on (0001) sapphire substrates. For growth duration of (**a**) 1 h, (**b**) 3 h, (**c**) 6 h, XRD spectra of the structures grown at 5 cm, 15 cm, 17.5 cm, 20 cm, 22.5 cm, 25 cm, and the insets of the enlarged spectra at position of 15 cm, 17.5 cm and 20 cm are presented alongside illustration of a reference pattern of the planes of crystallization for ZnO structures and sapphire substrate.

**Figure 5:**
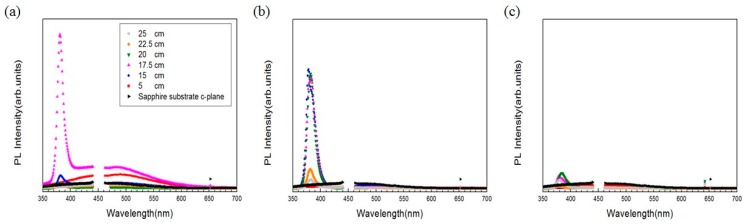
PL spectra of the ZnO micro/nanostructures grown on (0001) sapphire substrates excited with laser wavelength of 325 nm. For the growth duration of (**a**) 1 h, (**b**) 3 h, and (**c**) 6 h, PL spectra of the structures grown at 5 cm, 15 cm, 17.5 cm, 20 cm, 22.5 cm and 25 cm are depicted. The spectral results of 440 nm to 460 nm were omitted during data acquisition since the artificial peaks induced by the laser far exceeds the readable range of the photodetector.

**Figure 6:**
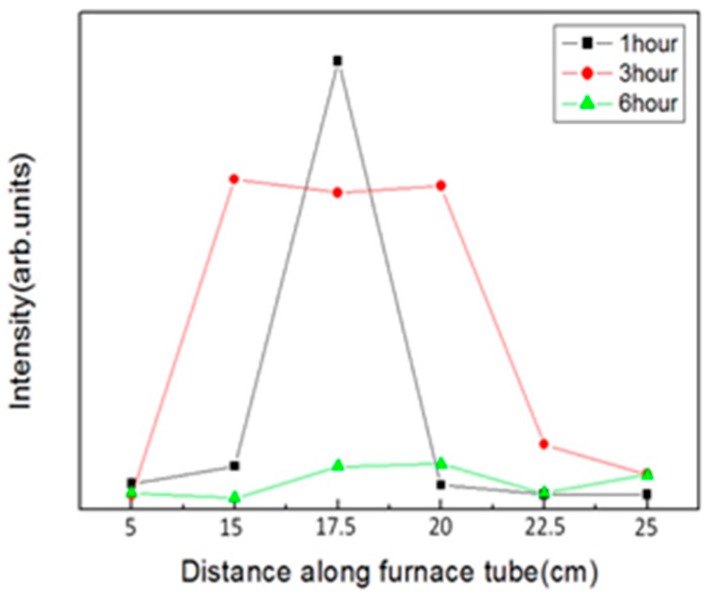
Spatial dependence of PL spectra of ZnO structures. For the growth duration of 1 h, 3 h and 6 h, peak PL of the structures grown at the position of 5 cm, 15 cm, 17.5 cm, 20 cm, 22.5 cm and 25 cm are illustrated. The excitation wavelength is 325 nm.

**Figure 7:**
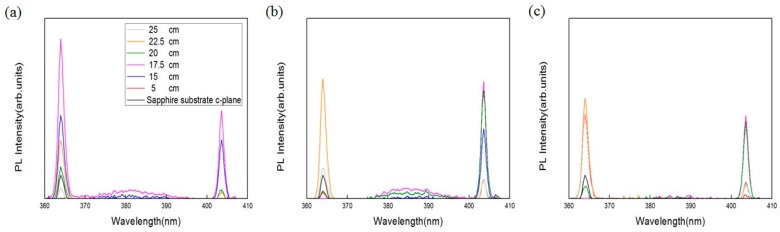
PL spectra of the ZnO micro/nanostructures grown on (0001) sapphire substrates excited with wavelength of 254 nm. For the growth duration of (**a**) 1 h, (**b**) 3 h, and (**c**) 6 h, PL spectra of the structures grown at 5 cm, 15 cm, 17.5 cm, 20 cm, 22.5 cm and 25 cm are depicted.

**Figure 8:**
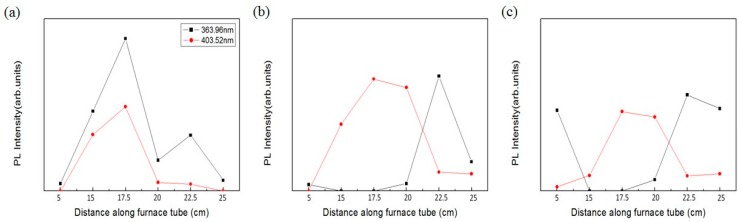
Spatial dependence of PL spectra of ZnO structures. For the growth duration of (**a**) 1 h, (**b**) 3 h and (**c**) 6 h, peak PL of the structures grown at the position of 5 cm, 15 cm, 17.5 cm, 20 cm, 22.5 cm and 25 cm are illustrated. The excitation wavelength is 254 nm.

**Figure 9:**
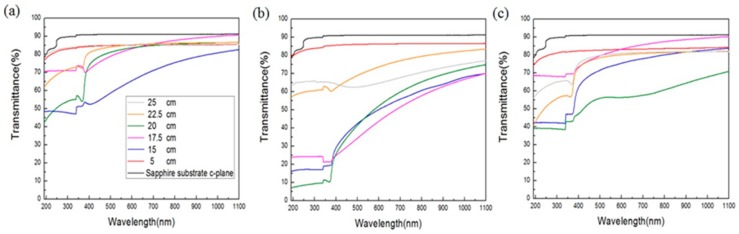
Transmission spectra of the ZnO structures on (0001) sapphire substrates. For growth duration of (**a**) 1 h, (**b**) 3 h and (**c**) 6 h, the structures grown at 5 cm, 15 cm, 17.5 cm, 20 cm, 22.5 cm and 25 cm are depicted.

Alongside the surface morphology presented by SEM images, XRD spectra illustrated in Figure 4 were acquired to examine the intrinsic crystalline properties, track the process of crystalization of the ZnO structures and elucidate the transitory formation of crystal planes associated with different growth duration and the temperature gradient. Also, PL spectra of the structures accompanied with typical emission peaks at around 380 nm were confirmed and are shown in Figure 5. To reveal any subtleties hidden in the PL profiles and analyze the position-dependent spectral change, the peak PL intensities of the structures grown with duration of 1 h, 3 h and 6 h are plotted as a function the distance along the furnace tube and are depicted in Figure 6. In addition to laser wavelength of 325 nm, the PL response of the structures under excitation of a UV lamp emitting photons with 254 nm wavelength was explored, and the PL spectra as well as their spatial dependence are shown in Figure 7 and Figure 8, respectively. Moreover, transmittance spectra of the structure were acquired and are presented in Figure 9.

## 4. Discussion

The methods of growing ZnO micro/nanostructures can be mainly categorized into chemical vapor transport (CVT) and physical vapor transport (PVT), which require only N_2_ gas for transportation of ZnO vapor [5,13]. However, it is recognizable that melting ZnO powder at 1975 °C is not an easy task for ordinary equipment to toggle and may have limited vapor for deposition. Past research suggested catalytic material such as graphite powder to drastically reduce the melting temperature by almost 2-fold [13]. Alternatively, the method of vapor-liquid-solid (VLS) that employs metallic films including Au, Ni, Cu and Co allows effective formation of crystalline structure on the substrates [16,17,18,19]. Additionally, a method, such as the utilization of organic ZnO precursor, Zinc acetylacetonate hydrate with low melting temperature at around 140 °C, has been exploited [8,14].

In this paper, the growth of ZnO structures on (1000) sapphire substrates in a low-temperature, vapor-trapped CVD was explored. Specifically, the dependence of the ZnO structures on the growth duration of 1 h, 3 h and 6 h, and spatially resolved temperature was investigated, and the structures were optically and structurally characterized, and the structural correlation with optical properties was analyzed.

Overall, in regard to the structural morphogenesis, the feature size of the structure enlarges as the growth duration increases, and the process of crystallization has a greater tendency to render a film-like structure. Spatially, regardless of growth duration, the feature sizes as well as the uniformity decrease and the structures become increasingly unorganized as the growth position gets closer to the center of the furnace, where maximal temperature occurs. For instance, the feature sizes of ZnO structures grown at 15 cm, shown in SEM images of Figure 2b,h,n, appear to be time-dependent, presenting larger feature size as the growth duration increases, and in particular, Figure 2h has a 3D granular pattern which is in high contrast when compared with the planar film-like structure of Figure 2n. Likewise, the morphology of the structures grown at the position of 17.5 cm has a similar trend as the position of 15 cm except for the unique grassy bush-like pattern associated with the grown duration of 6 h. Also, at the position of 20 cm, the structures are highly similar to that of the position of 15 cm in structural shape as well as the growth duration-dependent crystallization except with smaller density and low uniformity. A comparison between the position of 20 cm and 22.5 cm further confirms the aforementioned general remarks that Figure 2k is of similar structure as Figure 2j with lower density and smaller feature sizes, and the irregularly rocky pattern of Figure 2j is lost when the duration of growth time is extended from 3 h to 6 h.

To reveal the feature sizes and structural shapes in more details, SEM images with magnification of 50,000× were acquired and analyzed. Evidently, the position of 17.5 cm produces a unique stereoscopic ZnO structures. Specifically, the hexagonal structures with average sizes of 400 nm and 1 μm can be observed in Figure 3c,i, respectively, and Figure 3o resembles hexagonal structures packed by nanoblades with transitory sizes from approximately 500 nm down to the distal ends below 100 nm. At the position of 20 cm, the individual ZnO hexagonal structure of different feature sizes are presented in SEM images except in Figure 3j where the structure with feature sizes in the range of 600 nm to 700 nm appears to be agglomerated with rectangular pieces, and also, it is interesting to note that a tilted hexagonal structure with feature size of about 1 μm was captured in Figure 3p. To verify the sheer structures produced at the position of 22.5 cm and 25 cm, images with 50,000× magnification illustrate that the hexagonal structure of Figure 3l is slightly larger than that of Figure 3k. Surprisingly, Figure 3r is harnessed with hexagonal structure sitting on a film-like structure with high randomness in its detailed structural shapes. 

To ensure that the grown crystalline structure is, indeed, of ZnO material, XRD spectra were acquired and are presented in Figure 4. In detail, atop the strong peak intensity at 42° along the plane of (006) attributed to bare (0001) sapphire substrates, two main crystalline planes along (002) and (101) were found except at the position of 5 cm, 22.5 cm and 25 cm, where the surface profiles of the structures of SEM images are sporadic, irregular and random. 

In the structure of 15 cm with growth duration of 1 h, XRD signals along the planes of (100), (002) and (101) were found, and as the growth duration increases to 3 h, the signal at the plane of (101) intensifies, significantly exceeding that of sapphire substrate and the structures grown at other positions, corresponding to the increase of feature size presented in SEM images. Additionally, when the duration of growth extends to 6 h, the diffraction signal of 15 cm along all planes diminishes, which, in accordance with SEM images, implies a collapse of all crystalline planes into a concatenated morphology of mushroom heads. 

At the position of 17.5 cm, diffraction peaks along crystalline planes of (002) and (101) intensify gradually as the growth duration increases from 3 h to 6 h, signifying the temporally dependent enlargement of the structure. By contrast, the peak intensities of the position of 15 cm and 20 cm along (002) and (101) were found with growth duration of 3 h. Interestingly, at the position of 20 cm, though with weak XRD signals of all growth duration, the growth duration of 6 h exhibits weaker peak signals than that of 3 h, verifying the tendency of stereoscopic and planar thin film-like crystallization of the respective growth duration.

In addition to the structural properties examined by SEM and XRD, PL spectra of the structures excited with respective photon wavelength of 325 nm and 234 nm from He-Cd laser and UV lamp under ambient condition were acquired to determine how PL properties vary correlatively with the structural properties. 

PL spectra (325 nm-induced) of the structures grown for 1 h are illustrated in Figure 5a. While a wide distribution of weak PL intensities spanning across the range of visible wavelength was recorded with the structure grown at 5 cm, PL peaks of 380 nm associated with the position of 15 cm and 17.5 cm are especially pronounced, in which the one of 17.5 cm far exceeds that of 15 cm, flattens out from 400 to 500 nm, and decreases in intensity from 500 nm to 700 nm. In contrast, PL intensities associated with the position at 20 cm, 22.5 cm and 25 cm are weak, and no substantial signal can be observed. PL spectra of the structures grown with 3 h are presented in Figure 5b. At the position of 5 cm, a very weak and flat profile of intensity was recorded. Also, it is noted that the positions of 15 cm, 17.5 cm and 20 cm were observed with strong, overlapping PL peaks at 380 nm, whereas the position of 22.5 cm and 25 cm produce weak PL intensities. PL intensities with the growth duration of 6 h are presented in Figure 5c. At the position of 17.5 cm, 20 cm, 22.5 cm, 25 cm, weak intensity peaks of 380 nm were observed, and weak PL distribution over the range of visible wavelength was recorded.

Note that the changes of 325 nm-induced peak PL intensities as functions of growth duration and position, presented in Figure 6, are highly correlated with the results of XRD and SEM. At 15 cm, for instance, the peak PL intensity rises with the increase of growth duration from 1 h to 3 h, and then drops precipitously from 3 h to 6 h, which is in parallel with the variation of the XRD signal as well as the structural change presented in SEM images.

The strength of peak PL intensities of 20 cm as a function of growth duration is similar to that of 15 cm, where stereoscopic structure allows the highest PL compared with the thick film-like structure. Moreover, at the position of 17.5 cm, the peak PL intensity occurs at 1 h and drops diatonically for 3 h and 6 h, which implies a reduction in PL emissivity as the structure gets thicker and larger, whereas peak PL intensities flatten along the position of 15 cm to 20 cm for the growth duration of 3 h and 6 h. Other positions including 22.5 cm and 25 cm of all growth durations render low intensities, reflecting the null planes of crystallization of XRD spectra, and high randomness, low uniformity of the structural morphology of SEM images.

Most past research reported emission of PL at around 380 nm by excitation of the laser wavelength of 325 nm [8,14,15]. Herein, the PL response of the structures excited by the photon of wavelength of 254 nm from a UV lamp was characterized to analyze its dependence on the growth duration and spatially resolved temperature inside the furnace tube. As can be seen from Figure 7, all structures possess two dominant peaks of PL intensities solely at 363 nm and 403 nm. To conduct further analysis on how the peaking intensities vary as a function of position, 5 cm, 15 cm, 17.5 cm, 20 cm, 22.5 cm and 25 cm, peak intensities of both wavelength are plotted and are shown in Figure 8a–c for the respective growth durations of 1 h, 3 h and 6 h.

By carefully looking at Figure 8a, it can be seen that the intensities at 363.96 nm are consistently higher than those at 403.52 nm, both presenting dominant and minor peaks at 17.5 cm and 22.5 cm. Interestingly, for the growth duration of 3 h and 6 h, the profiles of peak intensities of 363.96 nm and 403.52 nm intersect at an extrapolated position equidistant between 20 cm and 22.5 cm as presented in Figure 8b,c; the variation of the peak intensities of 363.96 nm flattens from 5 cm to 20 cm; and the PL intensities of 403.52 nm and 363.96 nm peak at the position of 17.5 cm and 22.5 cm, correspondingly. However, one exception of the similarity from Figure 8b,c is the sample associated with growth duration of 6 h at the position of 5 cm, presenting substantial PL intensity considering the fact that null ZnO structures can be found in the corresponding SEM image, and our surmise is that this may be attributed to some random unintentional deposition of the remnant material. 

Contrary to the result of PL induced by 325 nm wavelength, the samples grown for 1 h at the position of 15 cm, 17.5 cm and 20 cm render substantial peak PL intensities of 363.96 nm, plummet precipitously and remain low for 3 h and 6 h. The entire trending behavior of the peaks of 403.52 nm is rather erratic, modulating the intensity high and low as the growth duration increases. The peak intensities of 403.52 nm of 22.5 and 25 cm remain low for all growth durations, whereas the peak intensities of 363.96 nm of 22.5 cm and 25 cm decrease diatonically toward the center of the furnace tube. Structurally, we found that PL of 363.96 nm associated with 22.5 cm intensifies as the feature size of the structure increases, and oppositely, the intensity of 25 cm swings up as the density and degree of randomness in structural shape increase, which is in sharp contrast to the PL of 380 nm. PL induced by 254 nm is found minimally correlated with structural morphology and XRD spectra.

Furthermore, the transmittance spectra were characterized using an UV-VIS spectrophotometer and are illustrated in Figure 9a–c for the growth duration of 1 h, 3 h and 6 h, respectively. Each profile of transmittance spectrum was a result of an average of three individual measurements with slight displacement of the chip samples. For the result of 1 h, the profiles of 5 cm and 25 cm flatten at around 82% of transmittance. The profiles of 15 cm, 17.5 cm and 20 cm have more pronounced variation in the range of 300 nm to 400 nm. Also, 15 cm and 17.5 cm have similar profile trends with 20% difference in transmittance, and the same behavior is also presented by 20 cm and 22.5 cm. 

With a growth duration of 3 h, the profile of 22.5 cm presents a gently declined slope toward the UV regime. Interestingly, the profiles of 15 cm, 17.5 cm and 20 cm depict parabolic variation between 375 nm and 1100 nm. Some conclusions can be drawn from the results of 3 h in comparison to those of 1 h. First, the profiles of 15 cm, 17.5 cm and 20 cm have very similar trends, and are lowered by about 45%; secondly, the profile of 22.5 cm is lowered only slightly and is accompanied by the reshaping of its contour; and third, the profile of 25 cm depicts a dipping well at 500 nm, and other position illustrate rather smooth and flat profiles. 

For the growth duration of 6 h, the structures of 15 cm, 17.5 cm, 20 cm, 22.5 cm and 25 cm exhibit pronounced sudden drops in transmittance at around 400 nm, of which the first three resemble the shape of ladders with different degrees of flatness, and the last two appear to be up-ramped curvy slopes toward the near-infrared (NIR) wavelength. A major remark of change from 3 h is the spike-up of the profiles of 15 cm, 17.5 cm and 20 cm. Structurally, the correlation of transmittance spectra with the structural results of SEM and XRD was less significant compared with PL. 

## 5. Conclusions

In this research, the growth of ZnO structures on (0001) sapphire substrates using Zinc acetylacetonate hydrate in a low temperature, vapor-trapped condition was attempted. SEM images, and XRD, PL, transmittance spectra were acquired to carry out structural and optical characterization, and investigate the correlation between optical and structural properties as well as the dependence of the structures on the growth duration, 1 h, 3 h and 6 h and spatially dependent temperature along the length of the quartz furnace tube.

Structurally, we found that the increase of growth duration corresponds well with the enlargement in feature size of the structure, the resultant crystallization has more tendency to produce a film-like structure, and regardless of growth duration, the density, feature sizes, uniformity and organization of the structures decrease as the growth position gets closer to the center of the furnace oven. Also, XRD spectra confirmed that stereoscopic structures enable more pronounced signs of crystallization than those of planar thin films. In the PL analysis, PL intensities induced by the laser wavelength of 325 nm are lowered as the structure gets thicker and larger; high randomness and low uniformity of the structures favor low PL emissivity; and the stereoscopic pattern allows the highest intensities, whereas the film-like structure emits poorly. Also, PL induced by 325 nm sheds better insight into structural correlation than that of the wavelength of 254 nm. Neither SEM images nor XRD spectra were highly correlated with the results of transmittance spectra; the jumps in several of the transmittance spectra are due to a measurement artifact from the change of lamp. As a whole, positions of 15 cm, 17.5 cm and 20 cm enable stronger signs of crystallization, stereoscopic structures and high PL response, providing the best combination of position and local temperature for the growth of ZnO micro/nanostructures in a low-temperature, air-trapped CVD system.
